# Human responses to nature- and culture-based non-clinical interventions: a systematised review

**DOI:** 10.1177/1757913920967036

**Published:** 2020-12-15

**Authors:** AJ Fairbrass, H Chatterjee, KE Jones, D Osborn

**Affiliations:** Centre for Biodiversity and Environment Research, Department of Genetics, Evolution and Environment, University College London, London, UK; Centre for Biodiversity and Environment Research, Department of Genetics, Evolution and Environment, University College London, London, UK; Centre for Biodiversity and Environment Research, Department of Genetics, Evolution and Environment, University College London, London, UK; Institute of Zoology, Zoological Society of London, London, UK; Department of Earth Sciences, University College London, London, UK

**Keywords:** culture, evidence, health, intervention, nature, review

## Abstract

**Aims:**

A wide range of non-clinical nature- and culture-based interventions for the treatment of health issues have been evaluated in evidence and systematic reviews. However, common outcomes of these interventions have not been identified and neuro-bio-psychosocial mechanisms underlying how these interventions impact health are not well understood. We conducted a systematised review and compared the evidence for human responses to nature- and culture-based non-clinical interventions for a range of health issues and assessed the proposed mechanisms and conceptual frameworks underlying these interventions.

**Methods:**

Comprehensive searches were conducted up to May 2018 in six bibliographic databases: Campbell Collaboration, Cochrane Library, Embase, Medline, Scopus and Web of Science. Studies included were evidence reviews or systematic reviews on any nature- or culture-based non-clinical intervention to improve the health of individuals.

**Results:**

A total of 60 reviews were included (33 of nature, 26 of culture, 1 of both) covering 1480 individual studies and trials. The most common review types were systematic (32), literature (22) and meta-analyses (6). Positive effects on mental health were reported for the majority of interventions, while other health outcomes such as immunity were not well represented in the review literature. A range of secondary outcomes were common to both nature- and culture-based interventions including psychological and emotional impacts, social interaction and relationship development, skills development, physical health benefits, and positive impact of the intervention environment. Only two reviews proposed conceptual frameworks, and the neuro-bio-psychosocial mechanisms that underpin the health changes were not clarified.

**Conclusion:**

Future research should focus on reviewing the evidence gaps for non-clinical nature- and culture-based interventions with an emphasis on implementing larger sample sizes, cohort and longitudinal studies, which deploy a wider range of mixed-methods, quasi-experimental and randomised control trials. There should also be agreement on terminology and developing conceptual frameworks to better understand the neuro-bio-psychosocial mechanisms underlying interventions.

## Introduction

Chronic non-communicable diseases are the leading cause of death globally.^1^ The costs of treating these diseases are high; in England, the treatment of long-term conditions now accounts for 70% of total health and social care spending.^2^ Non-clinical health interventions use activities rather than clinical services for prevention or treatment of chronic conditions and disease. They are formally delivered through partnerships between community-based organisations and health and social care providers, whereby healthcare professionals refer patients to non-clinical sources of support in the community to improve their health and wellbeing.^3^ As the demand on health and social care increases, non-clinical health interventions are being increasingly included in prevention and treatment plans of chronic health conditions.^3,4^

Nature- and culture-based non-clinical interventions are two broad categories of interventions that specifically use natural and cultural assets to deliver health and social care support. Nature-based interventions involve activities that change the environment in which people live, work, learn, recreate or heal to promote nature interactions. Alternatively, they change people’s behaviour through programmes or other means that involve engagement with nature.^5^ Culture-based interventions involve creative arts programmes (including visual and performing arts) and other cultural participation activities including engagement in festivals, museums, libraries, historic buildings and heritage sites.^4,6^ Examples of activities include care farming, environmental conservation, forest bathing (i.e. walking in, sitting in and/or viewing the forest),^7^ art therapies, book lending schemes and museum visits.

Multiple physical and mental health benefits are derived from engagement with nature- and culture-based activities, which lead to cost and efficiency savings for healthcare providers through avoided healthcare and medical costs.^3,4,6,8,9^ For example, economic analysis suggests that social prescribing provides a return on investment of £1.20–£11.55 for every £1 spent via mechanisms such as mitigating the negative impacts of social inequality and reducing the costly treatment needs of dementia.^4^ In terms of both nature- and culture-based interventions, the activity conducted provides participants with secondary health and wellbeing benefits, including increased physical activity; social interaction with therapists, carers and other participants; improved communication with caregivers; a sense of worth and purpose from contributing to a task; and skills development.^10,11^ In addition, the environment alone is proposed to provide added benefits such as a connection with natural and cultural heritage on top of the benefits derived from conducting the activity.^7,10,11^ Many of the outcomes identified for nature- and culture-based activities are shared across the two seemingly varied types of activities.^12^ These benefits contribute to a number of determinants of health such as social support and cohesion, personal growth and purpose in life,^13–15^ which may also link to the physiological mechanisms that support health.^16^

Increasing evidence suggests that non-clinical health interventions, including interventions using natural and cultural assets, are a valuable addition to treatment plans and relieve the burden of care from emergency and primary care services. These ‘upstream’ interventions have been shown to prevent conditions worsening and reduce demand for acute healthcare services.^3,4^ But efforts to mainstream these interventions in health service, social care or community support settings are held back by the lack of (1) theoretical or conceptual frameworks beyond those based on the wider determinants of health^13,14^ and (2) understanding of the mechanisms by which these interventions bring about benefit. It is likely that gaining an enhanced understanding of how different forms of engagement, and types of interventions or activities, bring about similar health outcomes will aid in the wider acceptance of such initiatives within society.^12^

Here we provide a systematised review of the field to examine how the existing evidence could help inform frameworks or mechanisms. To do this, we examined two classes of interventions (those involving activities based on nature or culture) and looked for commonalities in human responses to interventions, secondary outcomes and the proposed underlying mechanisms. We chose these classes of interventions as they have an extensive evidence base associated with them and share similar health outcomes from seemingly varied types of activities. Due to the increasing volume of review literature on this topic,^4,11^ we used a systematised review methodology to comprehensively compile the evidence from multiple reviews into one accessible and usable document.^17^

## Methods

### Search strategy

Comprehensive searches of the scientific literature were conducted in six electronic databases up to May 2018, including Campbell Collaboration, Cochrane Library, Embase, Medline, Scopus and Web of Science, and were restricted to English language publications that reported a review of primary studies. The search strategy consisted of the names of specific types of nature- and culture-based interventions such as ‘forest bathing’ and ‘art therapy’, as well as the broader terms ‘community referral’ and ‘social prescribing’, combined with the term ‘health’. Details of the Medline search strategy, which was adapted for all other electronic database searches, are provided in the Supplementary Information.

Non-clinical nature- and culture-based health interventions are typically delivered either by a professionally qualified therapist or by a professional trained in the intervention activity such as an artist, environmental conservationist or actor who may have no formal therapeutic qualification. To maximise the number of reviews that we included in this study, we did not limit our criteria of an eligible intervention to those delivered by a professional with a formal therapeutic qualification, although we acknowledge that this is a broader definition than is used by others.

### Study selection

All reviews of primary studies were eligible, including systematic reviews, meta-analyses, literature reviews, scoping and critical reviews, as defined by Grant and Booth.^18^ For a review to be included, the authors must have attempted to quantitatively or qualitatively synthesise the data from at least two primary studies. Both reviews with results pooled statistically in a meta-analysis and those with qualitative analyses were eligible for inclusion. Reviews must have addressed the impact on health of a nature- or culture-based intervention, excluded were reviews that addressed interventions defined as spiritual or sensory. For an intervention to be defined as nature-based, it had to involve changing the environment in which people live, work, learn, recreate or heal to promote nature interactions, and/or changing people’s behaviour through programmes or other means that involve engagement with nature.^5^ Interventions that used a typically outdoor activity such as running, but in an indoor environment such as a gym, were excluded. For an intervention to be defined as culture-based, we included any programme involving creative arts (including visual and performing arts) and other cultural participation activities including engagement in festivals, museums, libraries, historic buildings and heritage sites.^4^ Our list of eligible interventions was compiled by reviewing a number of recent reviews on this topic.^3,4,6,19^ The studies identified in the search were initially screened for relevance on the basis of their titles and abstracts. Subsequently, the full text of potentially relevant studies was assessed and studies were selected that satisfied the eligibility criteria. To summarise the exclusion criteria, reviews were excluded that did not synthesise results from at least two primary studies; did not report on a health outcome; and did not report on a nature- or culture-based intervention. Also excluded were studies that reviewed treatment programmes or methods of intervention implementation (rather than primary studies reporting on health outcomes) and/or that cited evidence without reference to primary studies (see the Supplementary Information for the Study Exclusion Criteria).

### Quality assessment and data extraction

The methodological quality of the reviews satisfying the eligibility criteria (see the Supplementary Information for the Study Exclusion Criteria) was assessed using the National Institute for Health and Care Excellence (NICE) guidelines for assessing systematic reviews and meta-analyses,^20^ as this method allows a consistent approach to assessing a broad range of review literature. Due to the nature of the review literature on nature- and culture-based non-clinical interventions, that is, the body of review literature is small and composed mainly of qualitative reviews or quantitative studies with small sample sizes, it was necessary to include reviews that scored poorly on the NICE quality assessment in order to draw more nuanced conclusions about the impact of nature- and culture-based interventions.

The NICE guidelines consist of seven criteria, five of which are rated as ‘yes’, ‘no’ or ‘unclear’, covering review characteristics such as literature search rigour, study quality assessment and reporting, and appropriateness and reporting of the review methods. We limited our use to these five criteria as their answers could be synthesised to produce an overall quality score for each review study. We considered that reviews following the Preferred Reporting Items for Systematic Reviews and Meta-Analyses (PRISMA) method satisfied the NICE criteria on appropriateness and reporting of methods. As an additional measure of methodological quality, we recorded whether the review protocol had been preregistered on a public repository such as the Cochrane Library. Criteria 1 (the review addresses an appropriate and clearly focused question that is relevant to the review question) and criteria 2 (the review collects the type of studies you consider relevant to the guidance review question) were retrospectively removed from our quality assessment as our exclusion criteria ensured that all included reviews satisfied these criteria.

Descriptive data were extracted using a standard form. Data collection included the following: general characteristics of the review (year of publication and type of review); clinical characteristics (age group, diagnosis of participants and type of intervention); methodological features (assessment methods used by the primary studies included in the review); results (number of primary studies included, review findings); suggested mechanisms of intervention actions; proposed theoretical or conceptual frameworks; research gaps; conclusions and recommendations for practice.

### Presentation of results

Evidence tables were produced to summarise the characteristics of the reviews and to synthesise the reported health and wellbeing outcomes. Health and wellbeing outcomes were classified following the International Statistical Classification of Diseases and Related Health Problems (ICD) 11th Revision.^21^

### Patient and public involvement

We did not involve patients or the public in this work.

## Results

**Figure 1:**
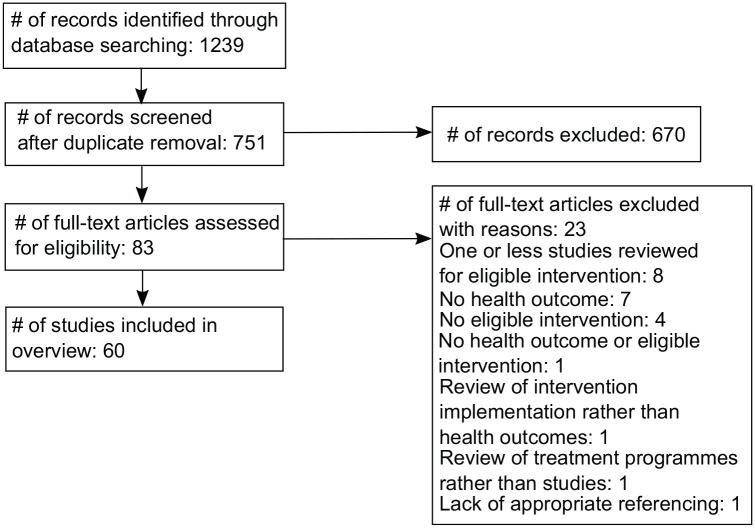
Study flow diagram

Literature searches up to May 2018 produced 751 studies of which 60 were included in this review (nature = 33, culture = 26, both = 1) reporting evidence from 1480 primary studies (see Table S1 and Supplementary Information). Figure 1 shows the flow of studies throughout the selection process. Twenty-three studies were excluded for the following reasons: eight reviewed only a single primary study; seven did not report on any health outcomes of the intervention; four did not report on an eligible intervention; one did not report on either a health outcome or an eligible intervention; one reviewed the implementation of interventions rather than health outcomes; one reported on treatment programmes rather than peer-reviewed studies; and one failed to cite references for statements made about health outcomes of interventions. A list of the 23 excluded studies and reasons for exclusion are available in the Supplementary Information.

### Description of the reviews

The reviews were published between 2005 and 2018, with more than half of the reviews (*n* = 36) published in 2014 or later. More than a quarter of the reviews (*n* = 17) were focused on populations of children and younger adults. General characteristics of the reviews are summarised in Table 1. Over 100 different measures representing the health status of participants were used in the studies covered (Tables S2 and S3).

**Table 1:** Descriptive characteristics of reviews of nature- and culture-based non-clinical interventions for health included in this review (*n* = 60)

	No. of studies
Group classification	
Children (<12 years)	8
Adolescents (13–18 years)	9
Adults (>18 years)	18
Undefined	35
Type of review	
Systematic review	28
Literature review	23
Meta-analysis	6
Cochrane systematic review	4
Scoping review	3
Critical review	1
Type of intervention	
*Culture*	
Visual arts	22
Music	6
Dance	3
Drama	2
Writing (including poetry, story-telling and journaling)	2
*Nature*	
Garden use	13
Horticulture	6
Care farming	5
Forest bathing	4
Outdoor exercise	3
Ecotherapy	2
Environmental conservation	1
Nature-assisted therapy	1

## Methodological Quality

Overall, the methodological quality of the reviews was moderate. The median quality score was 2 (interquartile range = 1.75–3) on a scale of 0 to 3. Thirteen reviews (22%) had major methodological flaws (a score of zero), while 22 reviews (37%) satisfied all the components of the quality appraisal (see Table S1 for the review ratings on the individual quality components). The most common flaw identified by the NICE quality appraisal was failure to assess and report the quality of studies included in the reviews (63% of reviews). In addition, 15 reviews (25%) either did not conduct a rigorous search or did not provide enough information to assess the rigour of the search strategy used. Thirteen reviews (22%) failed to provide an adequate description of the methodology used or the methods used were inappropriate to the review question. Eleven reviews (18%) preregistered their study protocol on a public repository.

## Effectiveness of Nature- and Culture-Based Non-Cinical Interventions

**Figure 2:**
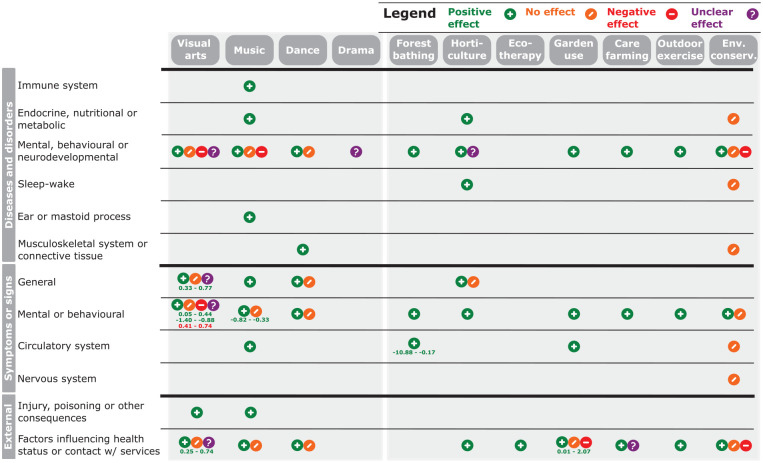
Findings in reviews of efficacy of culture-based (left-hand panel) and nature-based (right-hand panel) non-clinical interventions for health and wellbeing Results are limited to those reported by high-quality reviews (⩾2 quality score, Table S1). Significant results reported by meta-analyses are reported as the lower and upper confidence intervals of the effect size(s) and coloured to indicate the direction of effect reported (green = positive, red = negative). Health outcomes are grouped following the International Statistical Classification of Diseases and Related Health Problems (ICD) 11th Revision. See Table S4 for full results, including those reported in medium- and low-quality reviews (<2 quality score, Table S1), and Table S5 for mapping between health outcomes reported in reviews with the ICD classification system. ‘Env. conserv.’ refers to ‘Environmental conservation’ interventions.

Overall, the reviews tended to report positively on the effectiveness of nature- or culture-based non-clinical interventions, whereas only a small number of negative or unclear findings were reported (Figure 2, Table S4). Positive effects are those reported as improved health and wellbeing or reduced symptoms, whereas negative effects are those reported as worse health and wellbeing or increased symptoms. For example, the reduction of blood pressure by forest bathing^7^ is considered a positive effect, while the increase in psychosis symptoms by arts-based therapy^22^ is considered a negative effect. Outcomes related to mental, behavioural or neurodevelopmental diseases and disorders, symptoms or signs were reported for the majority of interventions. The least commonly investigated outcomes were related to immunity, the nervous system and ear health. Some specific health outcomes were more frequently associated with particular types of interventions in the review literature. For example, psychosis was only reviewed in relation to culture-based interventions, while stress was only reviewed in relation to nature-based interventions (Table S4). The majority of interventions investigated were focused on treatment rather than prevention of chronic conditions and disease. Only findings from high-quality reviews (⩾2 quality score, Table S1) are reported in Figure 2 to allow greater confidence in the results and conclusions.

## Secondary Health and Wellbeing Outcomes

**Figure 3:**
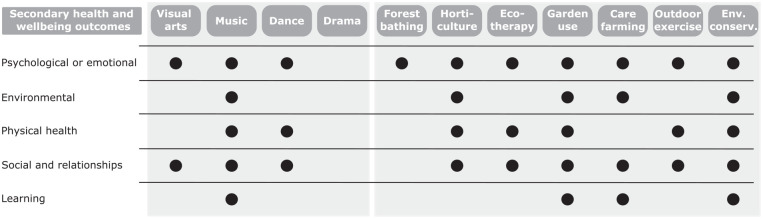
Potential secondary health and wellbeing outcomes proposed by reviews of culture-based (left-hand panel) and nature-based (right-hand panel) non-clinical interventions for health and wellbeing Only outcomes proposed by high-quality reviews (⩾2 quality score, Table S1) are reported. See Table S4 for full results, including those reported in medium- and low-quality reviews (<2 quality score, Table S1), and Table S6 for grouping of secondary health and wellbeing outcomes reported in reviews. ‘Env. conserv.’ refers to ‘Environmental conservation’ interventions.

Secondary health and wellbeing outcomes are effects that are hypothesised to play a role in achieving the primary outcome and include factors such as physical activity, social interaction and learning. A range of secondary health and wellbeing outcomes that may influence the effect of interventions were proposed in half of the reviews (*n* = 30), the majority of which were common across both nature- and culture-based interventions (Figure 3, Table S4). The most diverse group of secondary outcomes was of psychological or emotional nature, such as enjoyment and pleasure from taking part in the intervention and improved confidence. Outcomes related to social interaction and relationships were also commonly cited such as the development of relationships with carers and intervention providers. Physical health outcomes proposed included engagement in physical activity through the intervention and increased consumption of healthy food. The impact of the intervention environment was cited more commonly for nature-based interventions. Learning, including the development of knowledge and skills through the intervention activity, was also proposed for a number of intervention types.

## Mechanisms

Mechanisms are the underlying biological, physiological, psychological and neurological processes which explain how and why an intervention works, such as which neural pathways are involved when participants in music-based therapy are singing. These are in contrast to the outcomes of interventions, be they primary or secondary outcomes, which are the resultant effect(s) or impact(s) of the intervention, which are themselves the results of mechanisms. Two reviews proposed conceptual frameworks, including a review of the health impacts of environmental conservation activities^11^ and a review of the health and wellbeing impacts of gardening activities in schools.^10^ The model presented by Husk et al.^11^ includes a range of potential ‘mechanisms’: spirituality, change in personal/social identity, achievement/contribution, knowledge acquisition, social contact, being away from stressors, restoration/recuperation, enjoyment/pleasure, going into nature, self-confidence and physical activity. However, we refer to these as secondary outcomes (Figure 3, Table S4) rather than mechanisms which are the neuro-bio-psychosocial processes in the body that bring about health outcomes. The conceptual model proposed by Ohly et al.^10^ is composed of a suite of outcomes common to the other reviews in this study (Table S4), which are organised into the following categories: (1) physical and social aspects of school gardening, (2) factors influencing success and sustainability, and (3) intermediate to long-term final outcomes. Mechanisms are not proposed in this model, but in the text of the paper, the authors cite the Attention Restoration Theory as a potential mechanism which suggests that contact with nature can restore depleted ability to concentrate.

## Discussion

### Principal findings

In this review, we synthesised the evidence provided by the review literature about the efficacy of nature- and culture-based non-clinical health interventions for health and wellbeing. This topic has received increasing attention in recent years with over half of the reviews having been published after 2013. The earliest review that we identified was published in 2005, with the numbers increasing rapidly from 2014. This trend suggests that there is a need for regular reviews of this growing literature field to ensure that new evidence is synthesised frequently to inform health and social care policy and practice. We found a wide range of types of nature- and culture-based non-clinical health interventions that have been examined using a review approach, and reporting on their efficacy was predominantly positive. However, the neuro-bio-psychosocial mechanisms underlying the association between interventions and outcomes were not well-articulated in the review literature. Two reviews proposed conceptual models but these focused on outcomes rather than mechanisms, which highlights the need to better understand mechanisms. The quality of the included reviews was moderate with 15 reviews (25%) scoring poorly on the NICE quality appraisal and a substantial number of reviews (63%) failing to appraise and report the quality of their primary studies. The lexicon of non-clinical interventions is very complex and it is clear there is no agreed terminological or methodological framework. This makes evaluation difficult as each study tends to stand alone. It also makes learning and synthesis challenging. Nevertheless, some common health and wellbeing outcomes emerged, including psychological and emotional impacts, social interaction and relationship development, skills development, physical health benefits and positive impact of the intervention environment.

### Strengths and weaknesses of this study

Here we present the results of a systematised review by qualitatively compiling evidence from multiple reviews to provide an overview of what is currently known about the efficacy of nature- and culture-based non-clinical health interventions, the proposed outcomes elicited by these interventions and the existing knowledge gaps. This is the first time that the nature- and culture-based non-clinical health intervention review literature has been evaluated in this way. We restricted our search strategy to using scientific literature databases which will have restricted our included reviews to peer-reviewed publications. This potentially will have introduced publication bias to our results, particularly as grey literature dominates the field of non-clinical health interventions. For example, a recent UK government report on culture-based non-clinical health interventions has over 1000 citations, comprising a range of peer-reviewed intervention studies and grey literature.^4^ Due to the qualitative nature of most of the reviews included, we were unable to assess the risk of publication bias in this study. Publication bias is difficult to evaluate among reviews of non-randomised studies, and quantitative methods for assessing publication bias are not suitable with sample sizes of less than 10,^23^ of which our study includes only 6 reviews reporting quantitative meta-analyses. Eleven reviews had preregistered protocols which should have reduced the risk of publication bias for these reviews. We used strict inclusion criteria to ensure that the quality of evidence included in our review was high, demonstrated by three quarters of reviews scoring moderate to high on the NICE quality appraisal. This approach will have reduced the number of reviews included in this study. Our search strategy used the names for specific types of interventions chosen by reviewing recent reviews of the field;^3,4,6,19^ this approach will have limited our search terms to well-established types of interventions while more novel interventions were potentially missed. However, it is less likely that novel interventions would have amassed enough primary studies to have been examined using a review methodology. We made subjective choices about what constitutes a ‘nature’- and ‘culture’-based intervention which will have impacted the breadth of interventions satisfying our inclusion criteria; interventions classified as spiritual or sensory were excluded.

### Strengths and weaknesses in relation to other studies

Evidence on the efficacy of nature- and culture-based non-clinical health interventions has tended to be reviewed separately, for example, the role of nature-based interventions for mental health^3^ and the arts for health and wellbeing,^4^ highlighting the silos that exist in this research field. We are not aware of any studies to date that have specifically compared the review literature for nature- and culture-based non-clinical health interventions. By conducting this systematised review, we highlight trends in the review literature of specific intervention/health outcome combinations. For example, the majority of outcomes reported were related to mental health and there are a number of areas of physical health that have not been tackled by high-quality review studies. A surprising finding is the lack of high-quality review studies investigating the impact of nature-based interventions on diseases and disorders of the immune system given the evidence for links between exposure to natural environments and immunity.^24^ The application of statistical methods recently developed for quantitatively synthesising evidence of multiple reviews^25^ would be a useful avenue to develop the research presented here in the future.

### The meaning of the study: possible explanations and implications for clinicians and policymakers

In general, the vast majority of reviews reported positive health impacts of nature- and culture-based non-clinical health interventions, while a small number of meta-analyses reported significant positive effect sizes for some interventions, suggesting that these interventions may be an effective addition to patient treatment plans. These findings are particularly pertinent given recent policy shifts from the UK’s National Health Service (NHS) to incorporate social prescribing within their Universal Personalised Care model of healthcare delivery, as outlined in the NHS Long Term Plan.^26,27^ The recent rapid increase in the review literature suggests that there is increasing interest in the use of nature- and culture-based non-clinical health interventions in practice. However, the conceptual and theoretical frameworks underlying this research are not well developed and the underlying neuro-bio-psychosocial mechanisms are not well understood. This makes integrating these interventions into mainstream health and social care challenging. In concordance with previous reviews,^4^ our findings highlight a knowledge gap in relation to the use of non-clinical interventions for improving health through prevention as the majority of reviews covered treatment interventions. However, natural and cultural assets have been shown to be highly economically valuable in terms of their preventive health effects. For example, in England, it is estimated that access to greenspace could save £2.1 billion per year in health costs due to increased physical activity alone,^28^ while the arts, museums and heritage sites save the NHS around £700 million per year through reduced general practitioner (GP) visits and use of mental health services.^29^ Unfortunately, both natural and cultural public assets are under threat in the UK due to government underfunding.^9,30,31^ The degradation and loss of these assets will have considerable economic costs due to the lost preventive health services they provide.^32^ Several review authors highlighted the need to investigate the role of intervention characteristics^33^–^35^ and dose–response relationships^36^ on intervention efficacy to inform design guidelines and programmes for the delivery of interventions and this is an area of much needed research.

### Unanswered questions and future research

Future research of this kind should focus on reviewing the evidence gaps for non-clinical nature- and culture-based interventions with an emphasis on implementing larger sample sizes, cohort and longitudinal studies, deploying a wider range of mixed-methods, quasi-experimental and, finally, randomised control trials – but only when this later approach is appropriate in the more controlled circumstances. There must be a focus on improving the rigour of studies, and more use of quantitative as opposed to self-report measures (see Table S2 for the methods/outcome measures used by the primary studies reviewed). There should also be agreement on (1) methodological terminology, and (2) the need to develop conceptual frameworks so that a better understanding can be developed of the neuro-bio-psychosocial mechanisms underlying interventions. There are already well-tested models of health and wellbeing^13,14^ that can form the basis of this research. Without this mechanistic appreciation, it will be more difficult to design effective interventions that practitioners can apply with confidence and, consequently, more difficult for commissioning bodies to mainstream these kinds of interventions.

## Conclusion

Here we present the first systematised review of the health outcomes of nature- and culture-based non-clinical interventions. The evidence from the review literature suggests that these interventions deliver a wide range of positive health outcomes, and that inclusion of nature- and culture-based non-clinical health interventions in health and social care plans may be effective at improving the lives of sufferers of chronic health conditions. However, there is a lack of understanding of the neuro-bio-psychosocial mechanisms underlying the associations between interventions and human health, which impedes the quality of studies, evidence and uptake by health and social care providers. There are a number of health issues and interventions which are currently understudied in the review literature, which may reveal additional health benefits in the future. The use of nature- and culture-based interventions as preventive public health measures would be economically effective, but requires government commitment to maintain high-quality natural and cultural public assets. As the global prevalence of chronic health conditions increases, the maintenance of natural and cultural assets for public health must be an international priority.

## Supplemental Material

sj-docx-1-rsh-10.1177_1757913920967036 – Supplemental material for Human responses to nature- and culture-based non-clinical interventions: a systematised review Click here for additional data file. Supplemental material, sj-docx-1-rsh-10.1177_1757913920967036 for Human responses to nature- and culture-based non-clinical interventions: a systematised review by AJ Fairbrass, H Chatterjee, KE Jones and D Osborn in Perspectives in Public Health
