# Supplementary material for: Human responses to nature- and culture-based non-clinical interventions: a systematised review
Source: Perspect Public Health. 2020 Dec 15;142(3):149–57. doi: 10.1177/1757913920967036 (PMC9047098; doi:10.1177/1757913920967036)
Supplement: sj-docx-1-rsh-10.1177_1757913920967036 – Supplemental material for Human responses to nature- and culture-based non-clinical interventions: a systematised review [file sj-docx-1-rsh-10.1177_1757913920967036.docx]

**Table S1.** Characteristics of included studies and individual components of methodological quality, as measured by a shortened version of the NICE guidelines for assessing systematic reviews and meta-analyses. The individual components are: Q1. The literature search is sufficiently rigorous to identify all the relevant studies; Q2. Study quality is assessed and reported; and Q3. An adequate description of the methodology used is included, and the methods used are appropriate to the question. The quality score is calculated by summing the number of Yes (1), No (0) and Unclear (0) responses across the three components. Whether the review protocol was pre-registered with a public repository is also reported.

| **Reference** | **Intervention** | **Health outcome** | **Studies (n)** | **Population** | **Review type** | **Q1** | **Q2** | **Q3** | **Score** | **Pre-register** |
| --- | --- | --- | --- | --- | --- | --- | --- | --- | --- | --- |
| Al-Delaimy & Webb 2017 | Garden use | Dietary behaviour; Physical health and wellbeing | 36 | Undefined | Literature review | No | No | No | 0 | No |
| Alaimo et al. 2016 | Garden use | Dietary behaviour; Physical health and wellbeing | 53 | Undefined | Literature review | No | No | No | 0 | No |
| Annerstedt & Wahrborg 2011 | Nature-assisted therapy | Schizophrenia; Dementia; Cancer; Physical and mental health | 38 | Undefined | Systematic review | Yes | Yes | Yes | 3 | No |
| Artz et al. 2017 | Care farming | Physical and mental health | 19 | Children | Literature review | No | No | No | 0 | No |
| Attard & Larkin 2016 | Art | Psychosis | 18 | Adult | Literature review | Yes | Yes | Yes | 3 | No |
| Berezowitz et al. 2015 | Garden use | Dietary behaviour | 16 | Children; Adolescents | Systematic review | Yes | No | Yes | 2 | No |
| Best et al. 2015 | Art | Cancer | 4 | Undefined | Systematic review | Yes | Yes | Yes | 3 | No |
| Boehm et al. 2014 | Art | Cancer | 13 | Adolescent; Adult | Systematic review and meta-analysis | Yes | Yes | Yes | 3 | No |
| Bradt et al. 2015 | Dance | Cancer | 3 | Undefined | Cochrane systematic review | Yes | Yes | Yes | 3 | Yes |
| Bungay & Clift 2010 | Art | Mental health | 8 | Undefined | Literature review | No | No | No | 0 | No |
| Chancellor et al. 2014 | Art | Alzheimer's | 15 | Adults | Literature review | Yes | No | Yes | 2 | No |
| Cheung et al. 2016 | Drawing | Patient illness perception | 32 | Undefined | Scoping review | Yes | No | Yes | 2 | No |
| Connellan et al. 2013 | Art; Garden use | Mental health | 10 | Undefined | Systematic review | Yes | No | Yes | 2 | No |
| Coon et al. 2011 | Outdoor exercise | Mental wellbeing | 11 | Undefined | Systematic review | Yes | Yes | Yes | 3 | No |
| Davis et al. 2015 | Garden use | Dietary behaviour | 13 | Children; Adolescents | Literature review | Yes | No | Yes | 2 | No |
| Derman & Deatrick 2016 | Art | Cancer | 6 | Children; Adolescents; Adults | Literature review | Yes | Yes | Yes | 3 | No |
| Detweiler et al. 2012 | Horticulture; Garden use | Aging; Dementia | 40 | Adult | Literature review | Unclear | No | No | 0 | No |
| Drahota et al. 2012 | Music | Various | 85 | Adult | Cochrane systematic review | Yes | Yes | Yes | 3 | Yes |
| Broome et al. 2017 | Art | Dementia | 14 | Adults | Systematic review | Yes | No | Yes | 2 | No |
| Elsey et al. 2018 | Care farming | Physical and mental health | 38 | Undefined | Systematic review | Yes | Yes | Yes | 3 | Yes |
| Eum & Yim 2015 | Literature; Art; Music | Stroke | 23 | Undefined | Literature review | No | No | No | 0 | No |
| Flood & Phillips 2007 | Art | Aging | 6 | Adults | Literature review | No | No | No | 0 | No |
| Genter et al. 2015 | Horticulture | Physical health; Stress; Aging | 10 | Undefined | Systematic review | Yes | Yes | Yes | 3 | No |
| Gueu et al. 2010 | Art | Cancer | 17 | Adult | Literature review | Yes | No | Yes | 2 | No |
| Gladwell et al. 2013 | Outdoor exercise | Physical and mental health; Stress; Immune response | 16 | Undefined | Literature review | Unclear | No | No | 0 | No |
| Gonzalez & Kirkevold 2014 | Horticulture; Garden use | Dementia | 16 | Adults | Scoping review | Yes | No | Yes | 2 | No |
| Gorman & Cacciatore 2017 | Care farming | Grief | 8 | Undefined | Systematic review | Yes | No | Yes | 2 | No |
| Guitart et al. 2012 | Garden use | Physical and mental health; Dietary behaviour | 87 | Undefined | Systematic review | Yes | No | Yes | 2 | No |
| Hansen et al. 2017 | Forest-bathing | Physical and mental health | 64 | Undefined | Systematic review | Yes | No | Yes | 2 | No |
| Husk et al. 2016 | Environmental conservation | Physical and mental health | 28 | Undefined | Cochrane systematic review | Yes | Yes | Yes | 3 | Yes |
| Ideno et al. 2017 | Forest-bathing | Blood pressure | 15 | Undefined | Systematic review and meta-analysis | Yes | No | Yes | 2 | Yes |
| Kamioka et al. 2014 | Horticulture | Mental health; Dementia | 4 | Undefined | Systematic review | Yes | Yes | Yes | 3 | Yes |
| Kondo et al. 2018 | Outdoor activities | Stress | 43 | Undefined | Systematic review | Yes | No | Yes | 2 | No |
| Langellotto & Gupta 2012 | Garden use | Dietary behaviour | 20 | Children; Adolescents | Meta-analysis | Yes | No | Yes | 2 | No |
| Leckey 2011 | Art | Mental health | 11 | Adolescent; Adult | Literature review | Yes | No | Yes | 2 | No |
| Lutgens et al. 2017 | Art; Music | Psychosis | 7 | Undefined | Systematic review and meta-analysis | Yes | Yes | Yes | 3 | Yes |
| McCormack et al. 2010 | Garden use | Dietary behaviour | 4 | Undefined | Systematic review | Unclear | No | Yes | 1 | No |
| Nicklett et al. 2014 | Garden use | Physical health | 8 | Adults | Scoping review | Yes | No | Yes | 2 | No |
| Ohly et al. 2016 | Garden use | Dietary behaviour; Physical health and wellbeing | 40 | Children; Adolescents | Systematic review | Yes | Yes | Yes | 3 | Yes |
| Pederson et al. 2016 | Care farming | Mental health | 10 | Undefined | Literature review | No | No | No | 0 | No |
| Puetz et al. 2013 | Art | Cancer | 27 | Undefined | Systematic review and meta-analysis | Yes | Yes | Yes | 3 | No |
| Robinson-O'Brien et al. 2009 | Garden use | Dietary behaviour | 11 | Children; Adolescents | Systematic review | Yes | No | Yes | 2 | No |
| Ruddy & Milnes 2005 | Drama | Schizophrenia | 5 | Undefined | Cochrane systematic review | Yes | Yes | Yes | 3 | Yes |
| Soga et al. 2017 | Horticulture | Physical health and wellbeing | 22 | Undefined | Systematic review and meta-analysis | Yes | No | Yes | 2 | No |
| Song et al. 2016 | Forest-bathing; Ecotherapy | Physical wellbeing | 52 | Undefined | Literature review | Yes | No | Yes | 2 | No |
| Steigen et al. 2016 | Care farming | Mental health; Drug use | 25 | Undefined | Literature review | Yes | Yes | Yes | 3 | No |
| Strom et al. 2016 | Music | Dementia | 25 | Adults | Systematic review | Yes | Yes | Yes | 3 | No |
| Stuckey & Nobel 2010 | Various | Various | 68 | Adult | Literature review | Yes | No | Yes | 2 | No |
| Tielsch & Allen 2005 | Drawing | Mental health | 10 | Children; Adolescents | Literature review | Unclear | No | No | 0 | No |
| Tsunetsugu et al. 2010 | Forest-bathing | Blood pressure; Heart rate; Mental health; Stress; Blood sugar; Immune response | 25 | Undefined | Literature review | Unclear | No | No | 0 | No |
| Uttley et al. 2015a | Art | Mental health | 15 | Undefined | Systematic review | Yes | Yes | Yes | 3 | Yes |
| Uttley et al. 2015b | Art | Mental health | 11 | Undefined | Systematic review | Yes | Yes | Yes | 3 | No |
| Van Lith et al. 2013 | Art | Mental health | 23 | Undefined | Critical review | Yes | No | Yes | 2 | No |
| Whear et al. 2014 | Garden use | Dementia | 17 | Adults | Systematic review | Yes | Yes | Yes | 3 | Yes |
| Wilson et al. 2009 | Ecotherapy | Physical and mental health | 47 | Undefined | Literature review | Unclear | No | No | 0 | No |
| Wood et al. 2011 | Art | Cancer | 14 | Adult | Systematic review | Yes | Yes | Yes | 3 | No |
| Wright & Wadsworth 2014 | Horticulture | Aging | 108 | Adult | Literature review | Yes | No | Yes | 2 | No |
| York & Wiseman 2012 | Horticulture | Physical health and wellbeing | 4 | Undefined | Systematic review | Yes | No | Yes | 2 | No |
| Yotis 2006 | Drama | Schizophrenia | 45 | Undefined | Literature review | Unclear | No | No | 0 | No |
| Young et al. 2016 | Art | Dementia | 17 | Adults | Systematic review | Unclear | No | Yes | 1 | No |

**Table S2.** Assessment methods reported by the reviews included in this systematised review. See Table S3 for abbreviations.

| **Reference** | **Assessment methods** |
| --- | --- |
| Al-Delaimy & Webb 2017 | Not stated |
| Alaimo et al. 2016 | Not stated |
| Annerstedt & Wahrborg 2011 | AAMD Adaptive Behavior Scale for Children and Adults; ASIS; BDI; BPC; BSI; C-FS-EI; CMAI; CPSP; CRAVE; DCMS; DRIE; FSRQ; GESS; GS-ES; GSymI; HAM-A; HAM-D; IES; JEQ; MLoCS; PANSS; PARS; PDQ; Peer Experiences Questionnaire; POMS; PS-ES; QOLI; RCS; RSEI; SBS; SCL-90-R; SDSS-C; S-ES; RSES; SIP; SPPA; SR Y-OQ; SSSCA; SSTICS; TCS; Y-OQ; ZDI |
| Artz et al. 2017 | BASC; Self and parent rating scales; Social Responsiveness Scale; BPRS; PANSS; GSE; BBS; POMA |
| Attard & Larkin 2016 | Not stated |
| Berezowitz et al. 2015 | Questionnaires, interviews, observations, journals, taste tests. Academic outcomes were measured by science, math, and in one case reading achievement test scores. Science achievement was assessed using a science achievement test based on the Junior Master Gardener curriculum; math achievement was measured using the Texas statewide standardized test or the FCAT. The FCAT also assessed reading achievement. |
| Best et al. 2015 | Not stated |
| Boehm et al. 2014 | No stated |
| Bradt et al. 2015 |  |
| Bungay & Clift 2010 | WeMWBS; HADS; Interviews; Focus groups; Questionnaires |
| Chancellor et al. 2014 | Not stated |
| Cheung et al. 2016 | Patient drawings; Interviews; Questionnaires |
| Connellan et al. 2013 | Not stated |
| Coon et al. 2011 | Exercise induced feeling inventory; Feeling scale; Felt arousal scale; Rating of perceived exertion; Subjective vitality scale; Activation-deactivation adjective checklist; Physical activity enjoyment scale; PAS; NAS; RSES, POMS; Tension and effort stress inventory; Attention checklist; Recent life events questionnaire; Urine adrenaline; Urine noradrenaline; Urine cortisol; Blood pressure; Oxygen uptake; HR; Plasma beta-endorphin concentrations; General affect scale; Questionnaire designed to assess the restorative effect of physical activity; Air quality |
| Davis et al. 2015 | Questionnaires; Surveys; Observations; Recall workbooks; Teacher surveys; Lunchroom observation; BMI; Body fat; Waist circumference; Blood pressure |
| Derman & Deatrick 2016 | Validated Faces Scale; Emotional Response Checklist; Questionnaires; PedsQL; A quantitative behavioral rating scale measuring enhanced well-being (authors’ own); Patients’ artwork; Thematic and process analysis; Interviews; Written descriptions surrounding patients’ artwork. |
| Detweiler et al. 2012 | CMAI; Incident reports; Medication use; Surveys of staff and residents’ family members. |
| Drahota et al. 2012 | Not stated |
| Broome et al. 2017 | Interviews; Focus groups |
| Elsey et al. 2018 | Interviews; Photography; Focus groups; Participatory observation; Video recording and photographic method; Coping Strategies Scale; GS-ES; MMSE; The Barthel Index; SFS; 5-item Mental Health Inventory; Simplified Nutritional Appetite Questionnaire; Youth Self-Report; Utrecht Coping List; PANAS; Perceived Stress Scale; Therapeutic POMS questionnaire; CORE-OM; IPC LOC; WHOQOL-BREF; the Norwegian version of the QOLS; BDI; STAI; STAI-SS |
| Eum & Yim 2015 | Not stated |
| Flood & Phillips 2007 | A general health systems survey; Philadelphia Geriatric Center Morale Scale; UCLA Loneliness Scale; GDS; PIL; HIP |
| Genter et al. 2015 | Questionnaires; Interviews; Focus groups; Diaries; Photographs; SF-36; Life Satisfaction Index; Freuency and average number of days of at least half hour of moderate activity; Cortisol measured in saliva; Dutch version of PANAS; Body mass; Height; BMI; Systolic and diastolic blood pressure; Lung function; PSS; Social provisions scale |
| Gueu et al. 2010 | Interviews; Thematic analysis of images; Questionnaires; BFI; CRI; EACS; ESAS; ESI-R; FACT; FKV; HADS; QoL; MAC; SASB; POMS; SCL-90-R; SF-36; STAI; TSK; WHOQOL-BREF |
| Gladwell et al. 2013 | Not stated |
| Gonzalez & Kirkevold 2014 | Interviews; Questionnaire; Sleep logs; Observations of garden use; Sleep monitored using wrist actigraphs; CMAI; MMSE; AARS; MPES; The Fall Severity Score; Medication use; Incidents reports from staff; Inappropriate behaviour reported from relatives; Use of ‘intermediate-dose’ medications; Degree of independence in carrying out the activities; Field notes mapping the participants challenges in activities and mood; Hasegawa Dementia Scale (Revised version); Mapping of negative and positive emotions; Actilume-L; PSQI; DCMS; MMSE; ARS; Baumgarten, Becker and Gauthier’s checklist |
| Gorman & Cacciatore 2017 | Interviews; Questionnaires; Observations; Focus groups; RSES; POMS; Total Mood Disturbance Score; WeMWBS; Connor-Davidson Resilience Scale; the 2006/7 European Social Survey |
| Guitart et al. 2012 | Not stated |
| Hansen et al. 2017 | Psychological tests; Reactive oxygen metabolite (d-ROM) levels and biological antioxidant (BAPs) potentials associated with psychological stress; Pre-post HRV; NK activity; Self-reported pain, depression level and health related quality of life; Oxy-hemoglobin concentrations in the prefrontal cortex; Physiological measurements of central nervous activity; Autonomic nervous activity; Biomarkers reﬂecting stress response; Changes in cerebral activity; Pulse rate; Heart-rate variability; Arterial blood pressure; POMS; Autonomic and parasympathetic nervous system activity; Salivary alpha-amylase; Salivary cortisol levels; Cerebral Blood Flow (hemodynamics); STAI; Numbers of NK and T cells; Granulysin, perforin; Granzymes A/B expressing lymphocytes in blood samples; MDA concentrations; Cytokine production; Serum cortisol; Testosterone assay; Lymphocyte assay; Near-infrared spectroscopy measurements; Total hemoglobin and oxyhemoglobin concentrations; Blood pressure indicators; Cardiovascular disease-related pathological factors including endothelin-1, homocysteine, renin, angiotensinogen, angiotensin II, angiotensin II type 1 receptor, angiotensin II type 2 receptor, inﬂammatory cytokines interleukin-6 and tumor necrosis factor; Journalling; HAM-D; MADRS; Questionnaires; Actigraphy; Functional Assessment of Chronic Illness Therapy-Spiritual well-being; SF-36; Cancer Fatigue Scale; STAI; Blood glucose levels; BDI; Interviews; PSS; Ontario Health Study; Immunoglobulin A concentration in saliva; Pretest phenotype questionnaires; SBP; DBP; HR; Spiroergometry; Flow cytometry; NCQ; PSQ; ROS; SVS; Visual Analog Scale; NDI; EQ VAS; EQ index; MPQ; number of trigger points in posterior neck region; ROM of cervical spine; NRS Pain; CPGS; SF-36 BPS; ICOAP; Subjective Stress Scale (Stress); EID; PSS; PRS [post-test only]; Bispectral Index System value |
| Husk et al. 2016 | HR; Grip strength; Aerobic capacity; BMI; Weight; Body composition; Fexibility; Blood pressure; Balance; Waist/hip ratio; WEMWBS; ESS, adapted from the Osgood Semantic Difference Scale; RSES; POMS; Community cohesion scale; SF-36, SF-12; Scottish Physical Activity Questionnaire |
| Ideno et al. 2017 | Not stated |
| Kamioka et al. 2014 | AARS; MPES; DASS21; WBA; PWI; The Life Satisfaction Index-A Form; Revised UCLA Loneliness Scale; Lubben Social Network Scale; RSES; BDI; NCSE; MVPT; FIM |
| Kondo et al. 2018 | Anthropometric measurements of the CVS; Saliva; Blood; Urine; Actigraph; Electroencephalography; Alpha amylase; Cortisol (saliva and blood); Blood pressure; HR; Interbeat interval, LF/HF component; RR interval; SDNN; Cognitive function; rMSSD; Poincare plot (SD1); Sleep duration; VLF component; Oxygenated and total hemoglobin; TP; Adrenaline; Noraderaline; Estradiol; Progesterone; NK activity; Blood glucose; Total cholesterol; Triglycerides; Insulin; DHEA-S; NT-proBNP; CRP; Dopamine; Hemoglobin; Sleep-actigraph; Immunoglobulin A; Chromogranin A; HADS; STAI; TFOAS; NCPCT; BDS; ZIPERS; Overall Happiness Scale; Philadelphia Geriatric Center Positive and Negative Affect Rating Scale; PAAS; PAS; NAS; PANAS; POMS; SD; Stress-Refresh feeling test; Questionnaires; UWIST MACL; UWIST MACL, VAS; PRS; Restoration Outcome Scale; SMHSQ |
| Langellotto & Gupta 2012 | Not stated |
| Leckey 2011 | Questionnaires; Geriatric Depression scale; SF-12; Medical Outcomes Social Support Scale |
| Lutgens et al. 2017 | SANS |
| McCormack et al. 2010 | Behavioral Risk Factor Surveillance System questions; Nonquantitative food frequency questionnaire; Document reviews; Observations; Interviews; Surveys |
| Nicklett et al. 2014 | Dartmouth Primary Care Cooperative Functional Health Assessment Charts; Self-reported mobility impairments; Minimum Data Set for Physical Functioning Scale; Observations; Questionnaires; Modified Barthel Index; METs according to participant disability levels; Metabolic rate; HR; V02, HR (% maximum HR) |
| Ohly et al. 2016 | Interviews; Focus groups; Interviews; Observation; Concept maps; Children's drawings; Video and photography; Journals; Children's workbooks and work; Talk story (informal chats); Questionnaires; Event logs; BMI; Waist circumference; SBP; DBP; Urinary sodium; Urinary creatinine; Estimated salt intake; Physical activity; Total body fat; Vitamin A intake; Vitamin C intake; Fiber intake; CADET; Urine samples; Block Food Screener; Food preference assessment tool; surveys; Taste test; Self-efficacy instrument; KIDSCREEN-10; Quality of school life instrument; Youth Life Skills Inventory; Self-Report of Personality Scale |
| Pederson et al. 2016 | Observations; Interviews; GSE; Coping Strategies Scale; QOLS; STAI; BDI; AFI; PRestoreS; Brooding Subscale from Ruminative Response Scale; LRI-R; SOC – 13; PANAS-PA; PSS; TFI-CS |
| Puetz et al. 2013 | Not stated |
| Robinson-O'Brien et al. 2009 | Recall workbooks; Questionnaires; Interviews; Journals; Surveys; Focus groups; Multiple choice exams |
| Ruddy & Milnes 2005 | Psychotic Inpatient Proﬁle; Ward Atmosphere Scale; Psychiatric Outpatient Mood Scales; Standardised observation procedure of patient social interaction; Naturalistic observation of social interaction; Nurses observation scale for inpatient evaluation-30; Wing Scale of Schizophrenic Symptoms; Weschler Adult Intelligence Scale; Becker’s genetic analysis of the Rorschach; Draw-a-person Body Image Scale; Venables rating scale for activity withdrawal; Global assessment of illness; Rating of improvement; Quantitative features of performance on Rorscach tests; SANS; SAPS; RSES; FIS |
| Soga et al. 2017 | Not stated |
| Song et al. 2016 | Salivary and serum cortisol levels; HRV; Blood pressure; Pulse rate; Prefrontal cortex activity; Urine adrenaline; Blood glucose levels; NK activity; Oxyhemoglobin concentrations in the prefrontal cortex; Parasympathetic and sympathetic nervous activity; LF/HF ratio; Oxygenated hemoglobin levels; RR intervals |
| Steigen et al. 2016 | Video; Focus groups; Interviews; Questionnaires; Participatory observations; Diaries |
| Strom et al. 2016 | Not stated |
| Stuckey & Nobel 2010 | STAI; UWIST MACL; Cortisol levels; Visual analog scales; Interviews; SF-36; BAI; Mini-POMS; Derogatis Affects Balance Scale; Edmonton Symptom Assessment Scale |
| Tielsch & Allen 2005 | Assessment of characteristics of human figure drawings made by the children |
| Tsunetsugu et al. 2010 | HR; RR interval; Salivary cortisol and salivary IgA concentrations; Blood pressure; Prefrontal activity; Pulse rate; DBP; SBP; POMS; Blood glucose levels; NK activity; Immunoglobulin A, G and M levels; EEG; ECG; Absolute hemoglobin concentration determined using the TRS technique |
| Uttley et al. 2015a | PedsQL; Asthma module; BDI-II; SF-8; PCS-8; MCS-8; Apathy Scale (Japanese version); MMSE; WMS-R; GDS; Barthel Index; DBD; Zarit Caregiver Burden Interview; University of California, Los Angeles PTSD Reaction Index (DSM-IV Child Version); GSevI; SCL-90-R; Medical Outcomes Study SF-36; SCL-90-R; fMRI CBF and correlation with anxiety using CBF; POMS; EACS; ESAS; STAI; SCL-90; SASB; WHOQOL-BREF; CRI; EORTC QoL Questionnaire-BR23; PANAS; STAI; RSES; CFB (change from baseline); PedsQL; POMS; SCSI; PANAS; RSES |
| Uttley et al. 2015b | Not stated |
| Van Lith et al. 2013 | Interviews; Focus groups; Validated Progress Evaluation scale; RSES; Questionnaires; CORE-OM; SCL-90; IIP; SOC; HONOS; BPRS; SFS; IIP; SANS; PercQoL; BSI; CORE-OM; CORE-SF; BDI; WHOQOL; LSP; HADS; PQRST |
| Whear et al. 2014 | CMAI; Affect Rating Scale; Sleep measured with a wrist actigraph; Physical activity measured through observations and an ambulatory device; Frequency of medication use |
| Wilson et al. 2009 | Questionnaires; Diaries; Interviews; HR; Zuckerman Inventory of Personal Reactions and the Overall Happiness Scale; RSES; POMS; Tennessee Self-Concept Scale; Pedometers |
| Wood et al. 2011 | Questionnaires; Interviews; Patients’ artwork; Therapists’ narratives of sessions; Ttress markers in salivary samples; POMS; WHOQOL-BREF; EORTC QLQ; CRI; (ESAS) STAI; SCL-90-R; SF-36; HADS; BFI; EACS; POMS: expressions of spirituality inventory revised; MAC |
| Wright & Wadsworth 2014 | Not stated |
| York & Wiseman 2012 | Not stated |
| Yotis 2006 | Standardized psychosocial rating scales |
| Young et al. 2016 | Interviews; Diaries; Focus groups; Observations; NPI-Q; FACS; MMSE; CSDD; NPI Nursing Home Version; QoL-AD; Observed Emotion Rating Scale; Continuous time sampling using dementia care mapping codes; HDS; ACE-R; GDS; NPI; BADLS; Autobiographical fluency task; CBS for Quality of Life in Dementia; GHQ; ZBI; comprehensive neuropsychological test battery |

**Table S3.** Terminology and abbreviations used in Table S2.

| **Term** | **Abbreviation** |
| --- | --- |
| Activities of daily living | ADL |
| Addenbrooke Cognitive Examination | ACE-R |
| Adolescent Coping Orientation for Problem Experiences | A-COPE |
| Adult Self-Image Scale | ASIS |
| American Association on Mental Deficiency | AAMD |
| Apparent Affect Rating Scale | AARS |
| Attentional Function Inventory | AFI |
| Backward Digit Span | BDS |
| Beck Anxiety Inventory | BAI |
| Beck Depression Inventory | BDI |
| Beck Youth Inventories – Second Edition | BDI-II |
| Behavioural Assessment System for Children | BASC |
| Berg Balance Scale | BBS |
| Body mass index | BMI |
| Brief Fatigue Inventory | BFI |
| Brief Psychiatric Rating Scale | BPRS |
| Brief Symptom Inventory (Anxiety and depression subscale, Interpersonal and hostility subscales) | BSI |
| Bristol Activities of Daily Living Scale | BADLS |
| C-reactive protein | CRP |
| Cardiovascular system | CVS |
| Cerebral blood flow | CBF |
| Child and Diet Evaluation Tool | CADET |
| Children’s Physical Self-Perception | CPSP |
| Chronic Pain Grade Scale | CPGS |
| Clinical Outcome in Routine Evaluation–Outcome Measure | CORE-OM |
| Cohen Mansfield Agitation Inventory (short form) | CMAI |
| Comprehensive Review of Addiction Variables | CRAVE |
| Coping Resources Inventory | CRI |
| Cornell Scale for Depression in Dementia | CSDD |
| Cornell–Brown Scale | CBS |
| Culture-Free Self-Esteem Inventory | C-FS-EI |
| Dehydroepiandrosterone Sulfate | DHEA-S |
| Dementia Behaviour Disturbance Scale | DBD |
| Dementia Care Mapping Scale | DCMS |
| Depression Anxiety Stress Scale 21 | DASS21 |
| Diagnostic and Statistical Manual of Mental Disorders – Fourth Edition | DSM-IV |
| Diastolic blood pressure | DBP |
| Drinking Related Locus of Control | DRIE |
| Edmonton Symptom Assessment Scale | ESAS |
| Electrocardiography | ECG |
| Electroencephalography | EEG |
| Emergency room | ER |
| Emotional Approach Coping Scale | EACS |
| Emotional State Scale | ESS |
| Environmental Identity Scale | EID |
| Euro-Qual 5D-3L VAS | EQ VAS |
| Euro-Qual Index | EQ index |
| European Organisation for Research and Treatment of Cancer quality of life scale | EORTC QLQ (QOL) |
| European Organization for Research and Treatment of Cancer | EORTC |
| Expressions of spirituality inventory | ESI-R |
| Feeling inferior scale | FIS |
| Florida Comprehensive Achievement Test | FCAT |
| Focus of Attention Scale | TFOAS |
| Freiburger Fragebogen zur Krankheitsverarbeitung | FKV |
| Frequency of Self-Reinforcement Questionnaire | FSRQ |
| Functional Assessment of Cancer Therapy | FACT |
| Functional assessment of communication skills | FACS |
| Functional independence measure | FIM |
| Functional magnetic imaging | fMRI |
| General Health Questionnaire | GHQ |
| Generalised Expectancy for Success Scale | GESS |
| Generalised Self-Efficacy Scale | GS-ES |
| Generalized Self-Efﬁcacy measure | GSE |
| Geriatric Depression Scale | GDS |
| Global Severity Index | GSevI |
| Global Symptom Index | GSymI |
| Hamilton Anxiety | HAM-A |
| Hamilton Depression | HAM-D |
| Health of the Nation Outcomes Scale | HONOS |
| Heart rate | HR |
| Heart rate variability | HRV |
| Hierarchic dementia scale | HDS |
| Hospital Anxiety and Depression Scale | HADS |
| Hypochondriasis Scale Institutional Geriatric | HIP |
| Immunoglobulin A | IgA |
| Impact of Event Scale | IES |
| Internal, Powerful Others, and Chance orientations Locus of Control Scale | IPC LOC |
| Inventory of Interpersonal Problems | IIP |
| Inventory of Personal Reactions | ZIPERS |
| Jessor Expectancy Questionnaire | JEQ |
| Lancashire Quality of Life Profile | PercQoL |
| Life Regard Inventory Revised | LRI-R |
| Life Skills Profile | LSP |
| Low frequency/high frequency (LF/HF) ratio | LF/HF |
| Malondialdehyde | MDA |
| McGill pain questionnaire | MPQ |
| Measure of Intermittent and Constant Osteoarthritis Pain | ICOAP |
| Medical Outcomes Study Short-Form Health Survey | SF-36 |
| Menorah Park Engagement Scale | MPES |
| Mental Adjustment to Cancer scale | MAC |
| Metabolic equivalents | METs |
| Mini Mental State Examination Score | MMSE |
| Montgomery-Asberg Depression Rating Scales | MADRS |
| Motor-free visual perception test | MVPT |
| Multidimensional Locus of Control Scale | MLoCS |
| N-terminal pro b-type natriuretic peptide | NT-proBNP |
| Natural killer cell | NK |
| Nature Contact Questionnaire | NCQ |
| Neck disability index | NDI |
| Necker Cube Pattern Control task | NCPCT |
| Negative Affect Scale | NAS |
| Neurobehavioral cognitive status examination | NCSE |
| Neuropsychiatric Inventory | NPI-Q |
| Not reported | NR |
| Numeric Rating Scale for Pain | NRS Pain |
| Pediatric Quality of Life Scale | PedsQL |
| Perceived Restorativeness Scale | PRestoreS |
| Perceived Restrictiveness Scale | PRestrictS |
| Perceived Stress Questionnaire | PSQ |
| Perceived Stress Scale | PSS |
| Personal Wellbeing Index | PWI |
| Personality Diagnostic Questionnaire | PDQ |
| Phobic Avoidance Rating Scale | PARS |
| Physical Activity Affective Scale | PAAS |
| Physical Self-Efficacy Scale | PS-ES |
| Pittsburg Sleep Quality Index | PSQI |
| Poincare plot | SD1 |
| Positive Affect Scale | PAS |
| Positive and negative affect scale | PANAS |
| Positive and negative Affect Schedule –Positive Affect subscale | PANAS-PA |
| Positive and Negative Symptom Scale | PANSS |
| Post Traumatic Stress Disorder Index | PTSD-I |
| Post-traumatic stress disorder | PTSD |
| Profile of Mood States | POMS |
| Purpose in Life Scale | PIL |
| Quality of life | QoL |
| Quality of Life Alzheimer’s Disease | QoL-AD |
| Quality of Life Inventory | QOLI |
| Quality of Life Scale | QOLS-N |
| Rapid Scaling Technique | PQRST |
| Relationships Change Scale | RCS |
| Restorative Outcome Scale | ROS |
| Root mean squared of successive differences | rMSSD |
| Rosenberg Self-Esteem Inventory | RSEI |
| Rosenberg Self-Esteem Scale | RSES |
| RR interval | RR |
| Scale for Assessment of Negative Symptoms | SANS |
| Scale for Assessment of Positive Symptoms | SAPS |
| Schoolagers Coping Strategies Inventory | SCSI |
| Self-Efficacy Scale | S-ES |
| Self-Perception Profile for Adolescents | SPPA |
| Self-reported Youth Health Outcome Questionnaire | SR Y-OQ |
| Semantic Differential | SD |
| Semantic Differential Scale of Self-Concept | SDSS-C |
| Sense of Coherence Scale | SOC |
| short form of CORE-OM | CORE-SF |
| Short Form questionnaire-12 items | SF-12 |
| Short Form questionnaire-8 items | SF-8 |
| Short Form questionnaire-8 items (SF-8) – Mental component | MCS-8 |
| Short Form questionnaire-8 items (SF-8) – Physical component | PCS-8 |
| Short Form-36 Bodily Pain Scale | SF-36 BPS |
| Sickness Impact Profile | SIP |
| Social Behaviour Scale | SBS |
| Social Functioning Scale | SFS |
| Social Support Scale for Children and Adolescents | SSSCA |
| Spielberger State-Trait Anxiety Index | STAI |
| Spielberger State-Trait Anxiety Index - state subscale | STAI-S |
| St Mary's Hospital Sleep Questionnaire | SMHSQ |
| Standard deviation of normal-to-normal intervals | SDNN |
| State-Trait Anxiety Inventory – State Subscale | STAI-SS |
| Structural Analysis of Social Behavior | SASB |
| Subjective Scale to Investigate Cognition in Schizophrenia | SSTICS |
| Subjective Vitality Scale | SVS |
| Symptom Checklist Revised | SCL-90-R |
| Symptom-Checklist-90 | SCL-90 |
| Systolic blood pressure | SBP |
| Tests of Everyday Attention | TEA |
| The Multi Observational Scale for the Elderly | MOSES |
| The Rivermead Behavioural Memory Test | RBMIT |
| Therapeutic Factors Inventory Cohesiveness Scale | TFI-CS |
| Timeresolved spectroscopy | TRS |
| Tinetti Performance Oriented Mobility Assessment | POMA |
| Total power | TP |
| Trierer Saklen zur Krankheitsverarbeitung | TSK |
| Trust and Cooperation Scale | TCS |
| University of Wales Institute of Science and Technology Mood Adjective Checklist | UWIST MACL |
| Very low-frequency | VLF |
| Visual Analog Scale | VAS |
| Warwick-Edinburgh Mental Well-Being Scale | WEMWBS |
| Wechler Memory Scale revised. | WMS-R |
| Work Behavior Assessment | WBA |
| World Health Organization | WHO |
| World Health Organization’s Quality of Life Instruments | WHOQOL-BREF |
| Youth Outcome Questionnaire | Y-OQ |
| Zarit Burden Interview | ZBI |
| Zung Depression Inventory | ZDI |

**Table S4.** Findings in reviews of efficacy of culture and nature-based non-clinical interventions for health and well-being. Reference numbers refer to the included studies listed in the Supplementary Information. FV denotes fruit and vegetables. QoL denotes quality of life. * denotes finding of high-quality reviews (≥2 quality score, Table S1). Meta-analyses results are report as [effect size, lower confidence interval, upper confidence interval].

| **Intervention** | **Positive effect** | **No effect** | **Negative effect** | **Unclear effect** | **Secondary health and well-being outcomes** |
| --- | --- | --- | --- | --- | --- |
| *Culture* |  |  |  |  |  |
| Visual arts | Depression14*, 24*, 1, 41* (post-treatment) [0.23, 0.05, 0.40], Anxiety8* [-1.1, -1.40, -0.88],14*, 17*, 24*, 41* (post-treatment) [0.28, 0.11, 0.44], 51*, Psychological and social recovery53*, Fear17*, Pain17*, 41* (post-treatment) [0.54, 0.33, 0.75] , 41* (during follow-up) [0.59, 0.42, 0.77]Collaborative behavior17*, Communication17*, Mental health and well-being22, 35*, 11, 52*, Medication use22, 35*, Coping24*, 56*, 51*, QoL22, 24*, 41* (post-treatment) [0.5, 0.25, 0.74], 11, 56*, 51*, Personal growth24*, Social interaction11, 22, 24*, 12*, 10*, Self-esteem11, 12*, 22, 51*, Attention12*, 60, Pleasure12*, Neuropsychiatric symptoms12*, Health11, Empowerment11, Physical health22, Life satisfaction22, Problem-solving ability22, Creativity22, Own health perception11, 22, Doctor visits22, Psychosis5*, Fatigue56*, Tiredness56*, Mood51*, Trauma51*, Distress51*, Dementia symptoms10*, 60, Aggressiveness10*, Self-expression10*, Memory60, Communication60, Screening for depression and anxiety in school-ages children49, Patient illness perception13*, | Depression8* [-0.3, -0.60, 0.00], 41* (during follow-up) [-0.09, 0.42, 0.22], QoL8* [0.15, -0.09, 0.40], 41* (during follow-up) [0.22, 0.09, 0.54], Fatigue41* (post-treatment) [0.16, 0.04, 0.37], Psychosis symptoms36* [-0.14, -0.78, 0.50], Long-term psychosis36*, Suffering of cancer patients7*, Cognition51*, Anxiety 41* (during follow-up) [0.08, 0.26, 0.42], | Psychosis5*, 36* [0.57, 0.41, 0.74], Mental health52* | Pain8*, Functional assessment8*, Coping8*, Mood8*, Psychosis5* | Relaxation8*, stimulation8*, comunication8*, emotional expression8*, making art can produce a state called “ﬂow”, a state of intense concentration, satisfaction, and enjoyment11, recalibrating identity56*, a welcoming environment where participants feel valued60, social interaction5*,13*, Attention restoration13* |
| Music | Psychosis 36* [-0.58, -0.82, -0.33], Schizophrenia3*, Anxiety3*, 19*, 21, 47*, 48*, Depression3*, 21, 47*, 48*, Physical activity21, Diastolic blood pressure19*, Respiration rate19*, Heart rate19*, Arterial blood pressure19*, Anxiolytic medication requirements19*, Dementia symptoms3*, 47*, Mood3*, 47*, Emotional state47*, Agitation47*, Disruptiveness47*, Aggression47*, Cardiovascular and pulmonary performance47*, Emotional balance48*, Pain control48*, Stress48*, Heart rate48*, Well-being48*, Tension48*, Serum cortisol levels48*, Hospital patients’ sense of control48*, Pain48*, Immunity48*, Psychological and physical symptoms of cancer48*, Alcohol and substance abuse3*, Behavioral disorders3*, Acquired brain injury3*, Youth delinquency3*, Mental retardation3*, Personality disorders3*, Hearing impairment3*, Attention3*, Heart rate3* | Long-term psychosis36*, Nutrition intake (singing administered before mealtimes)47*, Agitation47*, Self-esteem48* | Behavioural disturbances47* | Health outcomes of hospital patients19* | Improved skills36*, social contact36*, Improved relationship between patient and carer19*, music impacts autonomic nervous system and activate the limbic system47*, interaction with environment that lowers stress thresholds47*, auditory stimulation48*, calm neural activity that may help to restore effective functioning in the immune system partly via the actions of the amygdala and hypothalamus48* |
| Dance | QoL9*, Somatization9*, Vigor9*, Self-awareness48*, Shoulder range of motion48*, Body image48*, Word and listening recall48*, Problem solving48*, Self-esteem48*, Psychological well-being48* | Depression9*, Stress9*, Anxiety9*, Fatigue9*, Body image9* | None | None | Physical activity9*, social interaction9*, creative expression9* |
| Drama | Schizophrenia59 | None | None | Schizophrenia43* | None reported |
| Writing (including poetry, story-telling and journaling) | Neurosis21, Emotional and behavioral disorders21, Cognitive functions after stroke21, Problem perception21, Insight22, Life reviews22, Resolution of unresolved conﬂicts22 | None | None | None | None reported |
| *Nature* |  |  |  |  |  |
| Garden use | Attention14*, Stress14*, Social connection14*, 1, 28*, Vegetable intake16*, 34* [0.42, 0.07, 2.07], 37, Fruit intake 34* [0.08, 0.02, 0.12] FV intake6*, 37, 39*, 42*, Preference for vegetables16*, 34* [0.10, 0.01, 0.19], Attitudes towards FV16*, 39*, Willingness to taste FV16*, Knowledge of FV16*, 39*, Self-efﬁcacy to prepare/cook FV16*, Frequency of FV consumption1, Quantity of FV consumption1, Diversity of FV consumption1, Diversity of vegetable consumption6*, Physical activity1, 2, Self-rated health1, Weight1, Diabetes control1, Mental health1, 2, Diet2, Education about healthy food2, Physiological indicators2, Well-being2, Cognitive function2, Social cohesion2, Integration2, Empowerment2, Food security2, Functional limitation38*, Agitation54*, Nutrient intake39*, FV preference39*, Diastolic blood pressure39*, Academic performance6*, FV consumption6*, Health28*, Access to fresh food28*, Inappropriate behaviour26, Fall frequency26, Sleep26, Happiness26, Interest26 | Vegetable intake16*, Functional status38*, FV intake39*, Nutrient intake39*, FV preference39*, Well-being39*, Academic performance6*, FV consumption6*, Sadness26, Anger26, Anxiety26, Nutrition knowledge 34* [0.21, -1.19, 0.43], Preferences for fruit 34* [-0.02, -0.20, 0.01] | Exposure to toxic soil contaminants2, Exposure to ingestion of pollutants and toxic substances2, Attitudes towards FV39*, Knowledge of FV39* | Physical and mental wellbeing54* | Improved nutrient intake and nutritional status39*, with positive effects on cognition6*, improve school environment thereby increasing attendance6*, improve attitude towards school6*, improved school performance and behaviour39*,skills development2, 6*, 39*, social interaction2, 39*, community connections2, contact with nature2, 39*, relaxation2, 39* restoration2, stimulation2, emotional attachment to environment2, garden provides space to practice behaviors and thought processes that are not practiced in the residential home54*, sun exposure improves sleep26, physical activity39*, growing and nurturing crops to grow food39*, cooperation and teamwork39*, connection with cultural heritage and local foods39*, supportive environment39*, gaining environmental knowledge and awareness39*, time for reflexion39*, feelings of ownership, responsibility and empowerment39*, improve attitudes towards food39*, achievement, satisfaction and pride39*, confidence and self—esteem39*, sense of purpose, feeling valued and belonging39*, experiencing success in the garden39*, social and cultural cohesion39* |
| Horticulture | Health44* [0.42, 0.36, 0.48], Depression44*, Anxiety44*, Stress44*, Mood disturbance44*, BMI44*, QoL44*, Sense of community44*, Physical activity levels44*, Cognitive function44*, Vegetable consumption34*, Stress18, 23*, Social connection23*, 58, Personal development23*, Contact with nature23*, Healthy aging23*, 57*, Pain18, Attention18 Vitamin D production18, Circadian rhythms18, QoL18, Agitation18, Aggression18, Medication intake18, 58*, Fall frequency18, Fall severity18, Behaviour18, 26*, 32*, Sleep duration26*, Sleep pattern26*, Positive affect26*, Cognition26*, Medication use26*, Engagement57*, Connection57*, Mental health32*, Well-being58*, Feelings of better physical health58*, Negative feelings and thoughts58*, Sense of personal agency58*, Maintenance and relearning of skills for people with neurological impairments58*, Sense of self, roles and status58* | Affect26* | None | Mental health32*, Dementia32* | Increased access to FV may increase FV consumption34*, 44*, social interaction44*, decrease hesitance to try new foods34*, physical activity44*,58, by nurturing the natural environment, people related more easily to the concept and act of self-nurturing58, skills development58, sun exposure improves sleep26, relaxation32* |
| Care farming | Depression27*, 40, Anxiety20*, 27*, 40, Positive affect20*, 40, Stress27*, Perceived stress40, Rumination40, Self-efficacy20*, 27*, 40, Coping40, 46*, Physical health4, Behaviour4, Emotions4, Mental health20*, 46*, Self-esteem20*, 27*, Mood20*, 27*, Medication use20*, Confidence20*, 27*, Vocational rehabilitation20*, Well-being20*, 27*, Self-perception20*, Social behaviours4, 20*, 27*, Tiredness20*, Independance20*, Healthy lifestyle20*, Happiness20*, 27*, Physical activity27*, 46, Motivation27*, Self-respect27*, Self-acceptance27*, Empowerment27*, Life satisfaction27*, Anger27*, Negative cognitions27*, Mastery46*, Structure46*, Meaningfulness46*, Dignity46*, Social gains46* | None | None | QoL20* | Social interaction40, being a colleague40, sharing a considerate relationship with the farmer40, being away from daily routines40, fascination with the intervention context (the farm)40, achievement and satisfaction20, belonging and non-judgement20, creating a new identity20, distraction20, feeling valued and respected20, feeling safe20, learning skills20, meaningfulness20, nurturing20, reflection20, stimulation20, structure20, understanding the self20, contact with animals46*, supportive natural environments46*, the service leader as a signiﬁcant important other46*, social acceptance and fellowship with other participants46*, meaningful and individually adapted activities in which mastery can be experienced46* |
| Forest-bathing | Parasympathetic nervous activity50, Sympathetic nervous activity50, Blood pressure29*, 50, Prefrontal activity50, Pulse rate31*, 50, Diastolic blood pressure31* [-1.75, -2.38, -1.13], 50, Systolic blood pressure (SBP) 31* [-3.15, -4.12, -2.18], 31* (SBP ≥ 130 mmHg) [-6.33, -9.35, -3.31], 31* (SBP <130) [-3.85, -5.53, -2.17], 31* (walking) [-3.48, -5.17, -1.80], 31* (non-walking) [-2.99, -4.17, -1.80], 31* (young males) [-2.53, -3.59, -1.48], 31* (middle-aged or older females) [-7.16, -10.88, -3.45], 50, Depression29*, 50, Anxiety50, Respiratory frequency50, Tension50, Anger50, Fatigue50, Confusion50, Vigor50, Salivary cortisol50, Blood glucose50, Hostility50, Liveliness50, Natural killer cell activity50, Heart rate29*, 31* [-3.84, -5.27, -2.40], 50, Relaxation29*, 45*, Substance abuse29*, Feeling of safety29*, Calm29*, Well-being29*, Pulse rate31* [-3.84, -5.27, -2.40] | Systolic blood pressure 31* (middle-aged or older males) [-4.27, -8.38, -0.17] | Pulse rate50, Prefrontal activity50 | None | Stimulation45* |
| Outdoor exercise | Stress33*, Revitalisation15*, 25, Engagement15*, 25, Self-esteem25, Tension15*, 25, Confusion15*, Anger15*, 25, Depression15*, 25, Energy15*, Post-exercise recovery25, Systolic blood pressure25, Diastolic blood pressure25, Sympathetic activation25, Adrenaline25, Noradrenaline25, Cortisol25, Natural killer cell activity25, Heart rate variability25, Well-being15*, Enjoyment15*, Satisfaction15*, Intention to repeat activity15* | None | Calmness15 | None | Stress reduction through attention restoration and cognitive function33*, social interaction33*, and physical activity33*, exposure to natural environment25, exposure to sunlight which increases vitamin D levels25 |
| Ecotherapy | Stress33*, 55, Health33*, Concentration55, Self-esteem55, Depression55, Aggression55, Positive affect55, Social cohesion55, Social skills55, Physical activity55, Mental health55, Hospital stays55, Pain control55, Headache frequency55, Digestive illnesses55, Sick calls55, Stress recovery55, Aggression55, Violence55, Concentration55, Mental fatigue55, Hostility55, Heart rate55, Focus55, Delay of gratification for females55, Symptoms of attention deficit disorders55, Happiness55, Perceived restoration55, Blood pressure55, Anger55, Tension55, Confusion55, Vigour55, Anxiety55 | Delay of gratification for males55 | None | None | Stress reduction through attention restoration and cognitive function33*, social interaction33*, and physical activity33*, physical activity55, stress reduction55, stimulation55 |
| Environmental conservation | Grip strength30*, Satisfaction with daily life30*, Mental health30*, Physical activity30* | Aerobic capacity30*, BMI30*, Weight30*, Body composition30*, Flexibility30*, Blood pressure30*, Balance30*, Waist/hip ratio30*, Well-being30*, Depression30*, Sleeping30*, Self-esteem30*, Mood30*, Social cohesion30* | Anxiety30*, Stress30* | Depression30*, QoL30* | Spirituality30*, change in personal/social identity30*, achievement/contribution30*, knowledge acquisition30*, social contact30*, being away from stressors30*, restoration/recuperation30*, enjoyment/pleasure30*, going into nature30*, self-confidence30*, physical activity30* |
| Nature-assisted therapy | Schizophrenia3, Dementia3, Depression3, Alcohol and substance abuse3, Mood3, Anxiety3, Behavioral disorders3, Acquired brain injury3, Youth delinquency3, Mental retardation3, Personality disorders3, Hearing impairment3, Attention3, Heart rate3 | Dementia3, Depression3 | None | Schizophrenia3 | Non reported |

##

**Table S5.** Classification of health and well-being outcomes reported by reviews (Table S4) following the International Statistical Classification of Diseases and Related Health Problems (ICD) 11th Revision. Outcomes are classified following the first and second level of the classification hierarchy. Outcomes are marked as ‘Not classified’ when a suitable classification was not identified in the ICD.

| **Outcome** | **Top level classification** | **Second level classification** |
| --- | --- | --- |
| Academic performance | 24 Factors influencing health status or contact with health services | Factors influencing health status |
| Access to fresh food | 24 Factors influencing health status or contact with health services | Factors influencing health status |
| Acquired brain injury | 22 Injury, poisoning or certain other consequences of external causes | Injuries to the head |
| Adrenaline | 05 Endocrine, nutritional or metabolic diseases | Endocrine diseases |
| Aerobic capacity | X Extension Codes | Dimensions of external causes |
| Affect | 21 Symptoms, signs or clinical findings, not elsewhere classified | Mental or behavioural symptoms, signs or clinical findings |
| Aggression | 21 Symptoms, signs or clinical findings, not elsewhere classified | Mental or behavioural symptoms, signs or clinical findings |
| Agitation | 21 Symptoms, signs or clinical findings, not elsewhere classified | Mental or behavioural symptoms, signs or clinical findings |
| Alcohol and substance abuse | 06 Mental, behavioural or neurodevelopmental disorders | Disorders due to substance use or addictive behaviours |
| Anger | 21 Symptoms, signs or clinical findings, not elsewhere classified | Mental or behavioural symptoms, signs or clinical findings |
| Anxiety | 06 Mental, behavioural or neurodevelopmental disorders | Anxiety or fear-related disorders |
| Anxiolytic medication requirements | Not classified | |
| Arterial blood pressure | 21 Symptoms, signs or clinical findings, not elsewhere classified | Symptoms, signs or clinical findings of the circulatory system |
| Attention | 06 Mental, behavioural or neurodevelopmental disorders | Neurodevelopmental disorders |
| Attention deficit disorder symptoms | 06 Mental, behavioural or neurodevelopmental disorders | Neurodevelopmental disorders |
| Attitudes towards fruit and vegetables | 24 Factors influencing health status or contact with health services | Factors influencing health status |
| Balance | 21 Symptoms, signs or clinical findings, not elsewhere classified | Symptoms, signs or clinical findings of the nervous system |
| Behaviour | 21 Symptoms, signs or clinical findings, not elsewhere classified | Mental or behavioural symptoms, signs or clinical findings |
| Behavioural disorders | 06 Mental, behavioural or neurodevelopmental disorders | |
| Behavioural disturbances | 06 Mental, behavioural or neurodevelopmental disorders | |
| Blood glucose | 21 Symptoms, signs or clinical findings, not elsewhere classified | Symptoms, signs or clinical findings of blood, blood-forming organs, or the immune system |
| Blood pressure | 21 Symptoms, signs or clinical findings, not elsewhere classified | Symptoms, signs or clinical findings of the circulatory system |
| Body mass index (BMI) | 05 Endocrine, nutritional or metabolic diseases | Nutritional disorders |
| Body composition | 24 Factors influencing health status or contact with health services | Factors influencing health status |
| Body image | 21 Symptoms, signs or clinical findings, not elsewhere classified | Mental or behavioural symptoms, signs or clinical findings |
| Brain injury | 22 Injury, poisoning or certain other consequences of external causes | Injuries to the head |
| Calm | 06 Mental, behavioural or neurodevelopmental disorders | Anxiety or fear-related disorders |
| Cancer symptoms | 21 Symptoms, signs or clinical findings, not elsewhere classified | General symptoms, signs or clinical findings |
| Cardiovascular and pulmonary performance | 21 Symptoms, signs or clinical findings, not elsewhere classified | Symptoms, signs or clinical findings of the circulatory system |
| Circadian rhythms | 07 Sleep-wake disorders | Circadian rhythm sleep-wake disorders |
| Cognition | 21 Symptoms, signs or clinical findings, not elsewhere classified | Mental or behavioural symptoms, signs or clinical findings |
| Cognitive function | 21 Symptoms, signs or clinical findings, not elsewhere classified | Mental or behavioural symptoms, signs or clinical findings |
| Cognitive function after stroke | 21 Symptoms, signs or clinical findings, not elsewhere classified | Mental or behavioural symptoms, signs or clinical findings |
| Collaborative behaviour | 21 Symptoms, signs or clinical findings, not elsewhere classified | Mental or behavioural symptoms, signs or clinical findings |
| Communication | V Supplementary section for functioning assessment | Generic functioning domains |
| Concentration | 21 Symptoms, signs or clinical findings, not elsewhere classified | Mental or behavioural symptoms, signs or clinical findings |
| Confidence | 21 Symptoms, signs or clinical findings, not elsewhere classified | Mental or behavioural symptoms, signs or clinical findings |
| Confusion | 21 Symptoms, signs or clinical findings, not elsewhere classified | Mental or behavioural symptoms, signs or clinical findings |
| Connection | 21 Symptoms, signs or clinical findings, not elsewhere classified | Mental or behavioural symptoms, signs or clinical findings |
| Contact with nature | 24 Factors influencing health status or contact with health services | Factors influencing health status |
| Coping | 06 Mental, behavioural or neurodevelopmental disorders | 6E40 Psychological or behavioural factors affecting disorders or diseases classified elsewhere |
| Cortisol | 05 Endocrine, nutritional or metabolic diseases | Endocrine diseases |
| Delay of gratification for females | Not classified | |
| Delay of gratification for males | Not classified | |
| Dementia | 06 Mental, behavioural or neurodevelopmental disorders | Neurocognitive disorders |
| Dementia symptoms | 06 Mental, behavioural or neurodevelopmental disorders | Neurocognitive disorders |
| Depression | 06 Mental, behavioural or neurodevelopmental disorders | Mood disorders |
| Diabetes control | 05 Endocrine, nutritional or metabolic diseases | Endocrine diseases |
| Diastolic blood pressure | 21 Symptoms, signs or clinical findings, not elsewhere classified | Symptoms, signs or clinical findings of the circulatory system |
| Diet | 24 Factors influencing health status or contact with health services | |
| Digestive illnesses | 13 Diseases of the digestive system | |
| Dignity | Not classified | |
| Disruptiveness | 21 Symptoms, signs or clinical findings, not elsewhere classified | Mental or behavioural symptoms, signs or clinical findings |
| Distress | 21 Symptoms, signs or clinical findings, not elsewhere classified | Mental or behavioural symptoms, signs or clinical findings |
| Education about healthy foods | 24 Factors influencing health status or contact with health services | Factors influencing health status |
| Emotional and behavioural disorders | 06 Mental, behavioural or neurodevelopmental disorders | |
| Emotional state | 21 Symptoms, signs or clinical findings, not elsewhere classified | Mental or behavioural symptoms, signs or clinical findings |
| Emotions | 21 Symptoms, signs or clinical findings, not elsewhere classified | Mental or behavioural symptoms, signs or clinical findings |
| Empowerment | 21 Symptoms, signs or clinical findings, not elsewhere classified | Mental or behavioural symptoms, signs or clinical findings |
| Energy | 21 Symptoms, signs or clinical findings, not elsewhere classified | Mental or behavioural symptoms, signs or clinical findings |
| Engagement | 21 Symptoms, signs or clinical findings, not elsewhere classified | Mental or behavioural symptoms, signs or clinical findings |
| Enjoyment | 21 Symptoms, signs or clinical findings, not elsewhere classified | Mental or behavioural symptoms, signs or clinical findings |
| Exposure to ingestion of pollutants and toxic substances | 24 Factors influencing health status or contact with health services | Factors influencing health status |
| Exposure to toxic soil contaminants | 24 Factors influencing health status or contact with health services | Factors influencing health status |
| Fall frequency | 21 Symptoms, signs or clinical findings, not elsewhere classified | Symptoms, signs or clinical findings of the nervous system |
| Fall severity | 21 Symptoms, signs or clinical findings, not elsewhere classified | Symptoms, signs or clinical findings of the nervous system |
| Fatigue | 21 Symptoms, signs or clinical findings, not elsewhere classified | General symptoms, signs or clinical findings |
| Fear | 21 Symptoms, signs or clinical findings, not elsewhere classified | Mental or behavioural symptoms, signs or clinical findings |
| Feeling of safety | 21 Symptoms, signs or clinical findings, not elsewhere classified | Mental or behavioural symptoms, signs or clinical findings |
| Feelings of better physical health | 21 Symptoms, signs or clinical findings, not elsewhere classified | General symptoms, signs or clinical findings |
| Flexibility | 15 Diseases of the musculoskeletal system or connective tissue | Soft tissue disorders |
| Focus | 21 Symptoms, signs or clinical findings, not elsewhere classified | Mental or behavioural symptoms, signs or clinical findings |
| Food security | 24 Factors influencing health status or contact with health services | Factors influencing health status |
| Frequency/quantity/diversity of fruit and/or vegetable consumption | 24 Factors influencing health status or contact with health services | Factors influencing health status |
| Functional limitation | 24 Factors influencing health status or contact with health services | Factors influencing health status |
| Fruit and vegetable intake | 24 Factors influencing health status or contact with health services | Factors influencing health status |
| Grip strength | X Extension Codes | Dimensions of external causes |
| Happiness | 21 Symptoms, signs or clinical findings, not elsewhere classified | Mental or behavioural symptoms, signs or clinical findings |
| Headache frequency | 08 Diseases of the nervous system | Headache disorders |
| Health | Not classified | |
| Health outcomes of hospital patients | Not classified | |
| Healthy aging | 21 Symptoms, signs or clinical findings, not elsewhere classified | General symptoms, signs or clinical findings |
| Healthy lifestyle | 24 Factors influencing health status or contact with health services | |
| Hearing impairment | 10 Diseases of the ear or mastoid process | Disorders with hearing impairment |
| Heart rate | 21 Symptoms, signs or clinical findings, not elsewhere classified | Symptoms, signs or clinical findings of the circulatory system |
| Heart rate variability | 21 Symptoms, signs or clinical findings, not elsewhere classified | Symptoms, signs or clinical findings of the circulatory system |
| Hospital stays | Not classified | |
| Hostility | 21 Symptoms, signs or clinical findings, not elsewhere classified | Mental or behavioural symptoms, signs or clinical findings |
| Illness perception | 21 Symptoms, signs or clinical findings, not elsewhere classified | General symptoms, signs or clinical findings |
| Immunity | 04 Diseases of the immune system | |
| Independence | 21 Symptoms, signs or clinical findings, not elsewhere classified | Mental or behavioural symptoms, signs or clinical findings |
| Inappropriate behaviour | 21 Symptoms, signs or clinical findings, not elsewhere classified | Mental or behavioural symptoms, signs or clinical findings |
| Insight | 21 Symptoms, signs or clinical findings, not elsewhere classified | Mental or behavioural symptoms, signs or clinical findings |
| Integration | 21 Symptoms, signs or clinical findings, not elsewhere classified | Mental or behavioural symptoms, signs or clinical findings |
| Intention to repeat activity | Not classified | |
| Interest | 21 Symptoms, signs or clinical findings, not elsewhere classified | Mental or behavioural symptoms, signs or clinical findings |
| Life reviews | Not classified | |
| Life satisfaction | 21 Symptoms, signs or clinical findings, not elsewhere classified | Mental or behavioural symptoms, signs or clinical findings |
| Liveliness | 21 Symptoms, signs or clinical findings, not elsewhere classified | Mental or behavioural symptoms, signs or clinical findings |
| Maintenance and relearning of skills for people with neurological impairments | Not classified | |
| Mastery | Not classified | |
| Meaningfulness | Not classified | |
| Medication use | Not classified | |
| Mental health | 06 Mental, behavioural or neurodevelopmental disorders | |
| Mental health and well-being | 06 Mental, behavioural or neurodevelopmental disorders | |
| Mental retardation | 06 Mental, behavioural or neurodevelopmental disorders | Neurodevelopmental disorders |
| Mood | 21 Symptoms, signs or clinical findings, not elsewhere classified | Mental or behavioural symptoms, signs or clinical findings |
| Mood disturbance | 21 Symptoms, signs or clinical findings, not elsewhere classified | Mental or behavioural symptoms, signs or clinical findings |
| Motivation | 21 Symptoms, signs or clinical findings, not elsewhere classified | Mental or behavioural symptoms, signs or clinical findings |
| Natural killer cell activity | 21 Symptoms, signs or clinical findings, not elsewhere classified | Symptoms, signs or clinical findings of blood, blood-forming organs, or the immune system |
| Negative cognitions | 21 Symptoms, signs or clinical findings, not elsewhere classified | Mental or behavioural symptoms, signs or clinical findings |
| Negative feelings and thoughts | 21 Symptoms, signs or clinical findings, not elsewhere classified | Mental or behavioural symptoms, signs or clinical findings |
| Neuropsychiatric symptoms | 06 Mental, behavioural or neurodevelopmental disorders | |
| Neurosis | 06 Mental, behavioural or neurodevelopmental disorders | Anxiety or fear-related disorders |
| Noradrenaline | 05 Endocrine, nutritional or metabolic diseases | Endocrine diseases |
| Nutrition intake | 24 Factors influencing health status or contact with health services | |
| Pain | 21 Symptoms, signs or clinical findings, not elsewhere classified | General symptoms, signs or clinical findings |
| Pain control | 21 Symptoms, signs or clinical findings, not elsewhere classified | General symptoms, signs or clinical findings |
| Parasympathetic nervous activity | 08 Diseases of the nervous system | Disorders of autonomic nervous system |
| Perceived restoration | 21 Symptoms, signs or clinical findings, not elsewhere classified | Mental or behavioural symptoms, signs or clinical findings |
| Perceived stress | 24 Factors influencing health status or contact with health services | Factors influencing health status |
| Personal development | 06 Mental, behavioural or neurodevelopmental disorders | |
| Personal growth | 06 Mental, behavioural or neurodevelopmental disorders | |
| Personality disorder | 06 Mental, behavioural or neurodevelopmental disorders | Personality disorders and related traits |
| Physical activity levels | 24 Factors influencing health status or contact with health services | Factors influencing health status |
| Physical and mental wellbeing | Not classified | |
| Physiological indicators | Not classified | |
| Pleasure | V Supplementary section for functioning assessment | WHODAS 2.0 36-item version |
| Positive affect | 21 Symptoms, signs or clinical findings, not elsewhere classified | Mental or behavioural symptoms, signs or clinical findings |
| Post-exercise recovery | Not classified | |
| Preference for vegetables | 24 Factors influencing health status or contact with health services | Factors influencing health status |
| Prefrontal activity | Not classified | |
| Problem perception | 21 Symptoms, signs or clinical findings, not elsewhere classified | Mental or behavioural symptoms, signs or clinical findings |
| Problem solving | Not classified | |
| Psychological and social recovery | 21 Symptoms, signs or clinical findings, not elsewhere classified | Mental or behavioural symptoms, signs or clinical findings |
| Psychosis symptoms | 06 Mental, behavioural or neurodevelopmental disorders | Schizophrenia or other primary psychotic disorders |
| Pulse rate | 21 Symptoms, signs or clinical findings, not elsewhere classified | Symptoms, signs or clinical findings of the circulatory system |
| Quality of life | 24 Factors influencing health status or contact with health services | Factors influencing health status |
| Relaxation | 21 Symptoms, signs or clinical findings, not elsewhere classified | Mental or behavioural symptoms, signs or clinical findings |
| Resolution of unresolved conflicts | 21 Symptoms, signs or clinical findings, not elsewhere classified | Mental or behavioural symptoms, signs or clinical findings |
| Respiration rate | 21 Symptoms, signs or clinical findings, not elsewhere classified | Symptoms, signs or clinical findings of the respiratory system |
| Respiratory frequency | 21 Symptoms, signs or clinical findings, not elsewhere classified | Symptoms, signs or clinical findings of the respiratory system |
| Revitalisation | Not classified | |
| Rumination | 21 Symptoms, signs or clinical findings, not elsewhere classified | Mental or behavioural symptoms, signs or clinical findings |
| Sadness | 21 Symptoms, signs or clinical findings, not elsewhere classified | Mental or behavioural symptoms, signs or clinical findings |
| Salivary cortisol | 05 Endocrine, nutritional or metabolic diseases | Endocrine diseases |
| Satisfaction | 21 Symptoms, signs or clinical findings, not elsewhere classified | Mental or behavioural symptoms, signs or clinical findings |
| Satisfaction with daily life | 21 Symptoms, signs or clinical findings, not elsewhere classified | Mental or behavioural symptoms, signs or clinical findings |
| Schizophrenia | 06 Mental, behavioural or neurodevelopmental disorders | Schizophrenia or other primary psychotic disorders |
| Self-acceptance | 21 Symptoms, signs or clinical findings, not elsewhere classified | Mental or behavioural symptoms, signs or clinical findings |
| Self-awareness | 21 Symptoms, signs or clinical findings, not elsewhere classified | Mental or behavioural symptoms, signs or clinical findings |
| Self-efficacy | 21 Symptoms, signs or clinical findings, not elsewhere classified | Mental or behavioural symptoms, signs or clinical findings |
| Self-efficacy to prepare/cook fruit and vegetables | 21 Symptoms, signs or clinical findings, not elsewhere classified | Mental or behavioural symptoms, signs or clinical findings |
| Self-esteem | 21 Symptoms, signs or clinical findings, not elsewhere classified | Mental or behavioural symptoms, signs or clinical findings |
| Self-expression | V Supplementary section for functioning assessment | Generic functioning domains |
| Self-perception | 21 Symptoms, signs or clinical findings, not elsewhere classified | Mental or behavioural symptoms, signs or clinical findings |
| Self-rated health | 21 Symptoms, signs or clinical findings, not elsewhere classified | General symptoms, signs or clinical findings |
| Self-respect | 21 Symptoms, signs or clinical findings, not elsewhere classified | Mental or behavioural symptoms, signs or clinical findings |
| Sense of community | 24 Factors influencing health status or contact with health services | Factors influencing health status |
| Sense of control | 21 Symptoms, signs or clinical findings, not elsewhere classified | Mental or behavioural symptoms, signs or clinical findings |
| Sense of personal agency | 21 Symptoms, signs or clinical findings, not elsewhere classified | Mental or behavioural symptoms, signs or clinical findings |
| Sense of self, roles and status | 21 Symptoms, signs or clinical findings, not elsewhere classified | Mental or behavioural symptoms, signs or clinical findings |
| Serum cortisol levels | 05 Endocrine, nutritional or metabolic diseases | Endocrine diseases |
| Shoulder range of motion | 15 Diseases of the musculoskeletal system or connective tissue | Soft tissue disorders |
| Sick calls | Not classified | |
| Sleep | 07 Sleep-wake disorders | |
| Sleep duration | 07 Sleep-wake disorders | |
| Sleep pattern | 07 Sleep-wake disorders | |
| Social behaviours | 21 Symptoms, signs or clinical findings, not elsewhere classified | Mental or behavioural symptoms, signs or clinical findings |
| Social cohesion | 21 Symptoms, signs or clinical findings, not elsewhere classified | Mental or behavioural symptoms, signs or clinical findings |
| Social connection | 21 Symptoms, signs or clinical findings, not elsewhere classified | Mental or behavioural symptoms, signs or clinical findings |
| Social gains | 21 Symptoms, signs or clinical findings, not elsewhere classified | Mental or behavioural symptoms, signs or clinical findings |
| Social interaction | 21 Symptoms, signs or clinical findings, not elsewhere classified | Mental or behavioural symptoms, signs or clinical findings |
| Social skills | 21 Symptoms, signs or clinical findings, not elsewhere classified | Mental or behavioural symptoms, signs or clinical findings |
| Somatization | 06 Mental, behavioural or neurodevelopmental disorders | Disorders of bodily distress or bodily experience |
| Stress | 24 Factors influencing health status or contact with health services | Factors influencing health status |
| Stress recovery | 24 Factors influencing health status or contact with health services | Factors influencing health status |
| Structure | Not classified | |
| Substance abuse | 06 Mental, behavioural or neurodevelopmental disorders | Disorders due to substance use or addictive behaviours |
| Suffering of cancer patients | 21 Symptoms, signs or clinical findings, not elsewhere classified | General symptoms, signs or clinical findings |
| Sympathetic activation | 08 Diseases of the nervous system | Disorders of autonomic nervous system |
| Sympathetic nervous activity | 08 Diseases of the nervous system | Disorders of autonomic nervous system |
| Systolic blood pressure | 21 Symptoms, signs or clinical findings, not elsewhere classified | Symptoms, signs or clinical findings of the circulatory system |
| Tension | 21 Symptoms, signs or clinical findings, not elsewhere classified | Mental or behavioural symptoms, signs or clinical findings |
| Tiredness | 21 Symptoms, signs or clinical findings, not elsewhere classified | Mental or behavioural symptoms, signs or clinical findings |
| Trauma | 22 Injury, poisoning or certain other consequences of external causes | |
| Vegetable intake | 24 Factors influencing health status or contact with health services | Factors influencing health status |
| Vigour | 21 Symptoms, signs or clinical findings, not elsewhere classified | General symptoms, signs or clinical findings |
| Violence | 21 Symptoms, signs or clinical findings, not elsewhere classified | Mental or behavioural symptoms, signs or clinical findings |
| Vitamin D production | 05 Endocrine, nutritional or metabolic diseases | Nutritional disorders |
| Vocational rehabilitation | 24 Factors influencing health status or contact with health services | Reasons for contact with the health services |
| Waist/hip ratio | 05 Endocrine, nutritional or metabolic diseases | Nutritional disorders |
| Weight | 05 Endocrine, nutritional or metabolic diseases | Nutritional disorders |
| Willingness to taste fruit and vegetables | 24 Factors influencing health status or contact with health services | Factors influencing health status |
| Word and listening recall | 24 Factors influencing health status or contact with health services | Reasons for contact with the health services |
| Youth delinquency | 24 Factors influencing health status or contact with health services | Factors influencing health status |

**Table S6.** Classification of secondary health and well-being outcomes reported by reviews (Table S4).

| **Secondary health and well-being outcomes** | **Classification** |
| --- | --- |
| Access to fruit and vegetables | Environmental |
| Achievement, satisfaction and pride | Psychological or emotional |
| Attention restoration | Psychological or emotional |
| Communication | Social and relationships |
| Connection with cultural heritage | Environmental |
| Contact with animals | Environmental |
| Contact with nature | Environmental |
| Cooperation and teamwork | Social and relationships |
| Creative expression | Psychological or emotional |
| Emotional expression | Psychological or emotional |
| Enjoyment/pleasure | Psychological or emotional |
| Experience of success | Psychological or emotional |
| Feelings of ownership, responsibility and empowerment | Psychological or emotional |
| Growing food | Environmental |
| Improved confidence | Psychological or emotional |
| Improved environment | Environmental |
| Improved knowledge and skills | Learning |
| Improved nutrient intake and nutritional status | Physical health |
| Improved relationship between patient and carer | Social and relationships |
| Music impacts autonomic nervous system and activate the limbic system | Physical health |
| Physical activity | Physical health |
| Recalibrating identity | Psychological or emotional |
| Relaxation | Psychological or emotional |
| Sense of purpose, feeling valued and belonging | Psychological or emotional |
| Social and cultural cohesion | Social and relationships |
| Spirituality | Psychological or emotional |
| Stimulation | Psychological or emotional |
| The service leader as a signiﬁcant important other | Social and relationships |
